# Seaweed exposure modulates Escherichia coli plasmid conjugation rate

**DOI:** 10.1099/mic.0.001622

**Published:** 2025-10-14

**Authors:** David Sünderhauf, Macaulay Winter, Jasmine Ramshaw, Emily M. Stevenson, Michiel Vos

**Affiliations:** 1 Environment and Sustainability Institute, University of Exeter, Penryn TR10 9FE, UK; 2 European Centre for Environment and Human Health, Environment and Sustainability Institute, University of Exeter Medical School, Penryn Campus, Penryn TR10 9FE, UK; 3 Department of Biological Sciences, Clemson University, Clemson, South Carolina, USA; 4 The Marine Biological Association of the UK, The Laboratory, Citadel Hill, Plymouth PL1 2PB, UK; 5 School of Biological and Marine Sciences, University of Plymouth, Plymouth PL4 8AA, UK; 6 Marine Ecology & Society, Plymouth Marine Laboratory, Prospect Place, West Hoe Plymouth PL1 3DH, UK

**Keywords:** antimicrobial-resistant (AMR), bacterial persistence, coastal zone, conjugation, *Escherichia coli*, plasmid transfer

## Abstract

Seaweeds are a common and diverse component of coastal ecosystems and are known to be associated with *Escherichia coli* due to faecal pollution. As a biotic substrate, beach-cast seaweed may affect bacterial physiology and thereby horizontal gene transfer (HGT). Here, we test how the presence of three distinct senescing seaweed species affects *E. coli* plasmid conjugation. We allow the IncP plasmid pKJK5 to conjugate while supplying a substrate of *Palmaria palmata* (dulse), *Ulva lactuca* (sea lettuce) or *Fucus serratus* (serrated wrack). The three seaweed species induce distinct conjugative behaviours in *E. coli*: *U. lactuca* has no significant impact relative to a plastic control, the presence of *F. serratus* results in undetectable levels of conjugation and *P. palmata* promotes conjugation in a density-independent manner. This study highlights how biotic interactions can influence survival, HGT and antibiotic resistance in a human pathogen.

## Introduction

*Escherichia coli* and other antimicrobial-resistant (AMR) bacteria are frequently found in coastal ecosystems due to sewage spills and agricultural run-off [1,2]. *E. coli* persistence in these environments can form a danger to human health due to acute infection risks [3,5] and via gut colonization by resistant strains [6], which may establish reservoirs of AMR [7]. In the human gut, AMR bacteria can disseminate antibiotic resistance genes to resident members of the microbiome and thus act as reservoirs of AMR genes [8]. Abiotic factors (e.g. UV [9], salinity [10] and temperature [11]) are known to affect *E. coli* survival and horizontal gene transfer (HGT), including conjugation [12], in different ways – even though underlying mechanisms and ecological impacts remain unresolved. Even less is known about how other organisms (live or dead) influence survival and HGT of bacteria in the environment.

Seaweeds are a species-rich group that can dominate coastal ecosystems and which have been found to be associated with *E. coli* [13,14]. Little is known about the possible impacts of exposure to seaweeds on bacterial conjugation. *E. coli* has been shown to grow on nutrients leaching out of different beach-cast seaweeds [15,16], and it is therefore possible that seaweeds could promote conjugation via stimulating growth [17] and/or biofilm formation [18]. For instance, conjugation in *Roseobacter* was found to increase in the presence of the unicellular algae *Emiliania huxleyi*, most likely due to increased bacterial attachment and cell-cell contact [19]. More generally, the ‘phycosphere’ has been proposed to form a hotspot for bacterial HGT [20]. However, seaweeds are also prolific producers of secondary metabolites, and seaweed extracts and molecules are well-known for their antibacterial activity [21,22]. Thus, seaweeds could negatively impact bacterial survival rates in some scenarios and/or elicit stress responses that in turn could influence conjugation rate.

We here expose *E. coli* strains carrying a conjugative tetracycline resistance-conferring IncP1 plasmid pKJK5::gfp to rehydrated seaweed (a proxy for senescing, beach-cast seaweed) and measure bacterial survival and plasmid transfer. We use three phylogenetically divergent seaweed species abundant on North-West European shores: serrated wrack *Fucus serratus* (*Phaeophyceae*), dulse *Palmaria palmata* (*Rhodophyta*) and sea lettuce *Ulva lactuca* (*Chlorophyta*). We find that *E. coli* AMR plasmid acquisition can be significantly influenced by seaweed species due to survival-dependent and survival-independent processes. Coastal species assemblages thus have the potential to influence both the density and antibiotic resistance of human-associated bacteria.

## Methods

### Seaweeds, strains and plasmids

*F. serratus*, *P. palmata* and *U. lactuca* seaweeds were collected in July 2022 at Castle Beach, Falmouth, Cornwall, UK (50.1479° N 5.0567° W). Attached organisms were removed, and seaweeds were transported to the lab in sterile plastic bags within 1 h of collection. Seawater was collected and transported back in a sterile vessel at the same time. *E. coli* K12 MG1655::mCherry carrying pKJK5::gfp (*lacI*-repressible GFP and *aphA* inserted into *dfrA* of pKJK5 [23]) was used as a donor strain. *E. coli* K12 MG1655 carrying chromosomal *cat* chloramphenicol resistance and *mScarlet-I* fluorophore genes (*E. coli* K12 MG1655::cat), inserted by Tn7 transposition downstream of *glmS* (using plasmid pMRE-Tn7-155 [24]), was used as a recipient strain. In the donor strain, *gfp* expression of pKJK5::gfp was inhibited due to *lacI* integration into the K12 MG1655::mCherry genome. Frozen cultures of donor and recipient strains were suspended in Luria-Bertani (LB) broth (Fisher, UK) for 24 h at 28 ˚C and 180 r.p.m., where the donor strain was supplemented with 60 µg ml^−1^ tetracycline (Formedium, UK) to select for pKJK5::gfp maintenance.

### Conjugation assay

An ethanol-sterilized 10 mm diameter hole punch was used to produce uniform discs of each seaweed species. Plastic control discs were punched from polyethylene petri dish sleeves and sterilized via submersion in 70% ethanol solution (Fisher, UK). Each disc type was placed into separate wells of a 48-well plate (Fisher, UK), covered with the plate lid and incubated at 28 °C for 24 h to dry out (*N*=6). Afterwards, all wells were filled with 570 µl unsterilized seawater. An additional control included wells containing plastic discs submerged in 570 µl LB broth (*N*=3). Cultures of donor and recipient cells were washed three times in 0.85% NaCl and resuspended at equal OD (OD600=0.6). A volume of 15 µl of each strain was added to each well. As a control, two additional wells were used for each treatment which contained only donor or recipient cells, respectively (no transconjugants detected). The 48-well plate was then incubated statically at 28 ˚C for 48 h covered with the plate lid and parafilm to prevent evaporation. A control experiment showed that this prolonged incubation period was necessary to observe conjugation events. Before plating, adherent bacteria were dislodged into the planktonic phase by pulsing the culture medium using a pipette until no further visible change in substrate surface colour or texture was observed. A dilution series with 5 µl droplets (ranging from 10^0^ to 10^−7^) of each sample was plated on Chromocult^®^ Coliform agar at *t*_0_ and *t*_48_ (Sigma-Aldrich, USA) supplemented with 15 µg ml^−1^ tetracycline, or 6 µg ml^−1^ chloramphenicol (Sigma-Aldrich, USA), or with both antibiotics concurrently to select for cells containing pKJK5::gfp, recipients and pKJK5::gfp transconjugants, respectively. Transconjugant colonies were verified based on GFP expression assessed with a NightSea fluorescence lamp and Royal Blue bandpass filter. We calculated the conjugation rate as $conjugationrate(perday)=\frac{transconjugant\frac{cfu}{mL}}{donor\frac{cfu}{mL}\times recipient\frac{cfu}{mL}\times time[day]}$ [25]. While the convention is to report conjugation rates in hours, we opted to report this by day to make clear our experimental time frames and other caveats that should be considered when comparing conjugation rates to other literature values: where conjugation rate measures are accompanied by cell density changes, they may be inaccurate when compared with other literature values [26]. Comparisons between treatments remain robust and are mirrored in the population ratio of transconjugant fraction (transconjugants/recipients), the calculation of which is not dependent on consistent cell densities (Table S1, available in the online Supplementary Material). Additionally, donor and recipient strains are identical apart from their resistance and fluorophore gene tags. Therefore, differential transconjugant growth between treatments is unlikely to occur and skew relative conjugation rate estimates.

As our limit of detection of c.f.u. ml^−1^ within samples was 200 (1 colony in 5 µl undiluted sample), the limit of detection of conjugation rate was dependent on donor and recipient density: $\frac{200\frac{cfu}{mL}}{donor\frac{cfu}{mL}\times recipient\frac{cfu}{mL}\times 2days}$, or ~2 orders of magnitude above the inverse product of donor and recipient c.f.u. ml^−1^. C.f.u. ml^−1^ counts for all treatments and controls are available as a File S2Supplementary Data Sheet 1.

### Statistical analyses

Data and statistical analyses were carried out using R version 4.3.1 and RStudio version 2023.12.1+402. Differences in log_10_-transformed conjugation rate were assessed with pairwise estimated marginal means (EMMs) comparisons (‘emmeans’ package v1.8.6) using a linear model as input data (a Gaussian linear model with seaweed/substrate as an explanatory variable). Model residuals were checked using the ‘DHARMa’ package v0.4.5 [27]. Simulated residuals were visually inspected for deviations from uniformity, overdispersion and zero-inflation. Visual diagnostics showed acceptable residual behaviour for all models; both the Kolmogorov–Smirnov test and dispersion test returned non-significant results for models, suggesting no departures from ideal model assumptions. Graphs were produced using ‘ggplot2’ [28]. All instances of multiple testing were corrected for using false discovery rate adjustment. See Tables S2 and S3 and full text for test statistics.

## Results

### Senescing seaweeds affect *E. coli* conjugation rate through growth rate-dependent and growth rate-independent mechanisms

To test whether desiccated seaweed (a proxy for senescing, beach-cast seaweed) affects conjugation rate in *E. coli*, a donor strain carrying an IncP1 plasmid harbouring a tetracycline resistance gene (pKJK5::gfp) was incubated with a recipient strain in seawater to which three seaweed species, *P. palmata*, *U. lactuca* and *F. serratus*, were added. We started with equal concentrations (~4.4×10^7^ c.f.u. ml^−1^) of *E. coli* strains in 48-well plate wells containing unsterilized seawater and either a seaweed or control polyethylene plastic substrate. After 48 h, the samples were plated, and the total number of *E. coli* and the number of transconjugants were counted to calculate cell density and conjugation rate. A sterile high-nutrient (no substrate) control was used to verify that conjugation between *E. coli* strains could be detected in our experimental system (Fig. S1).

**Fig. 1.**
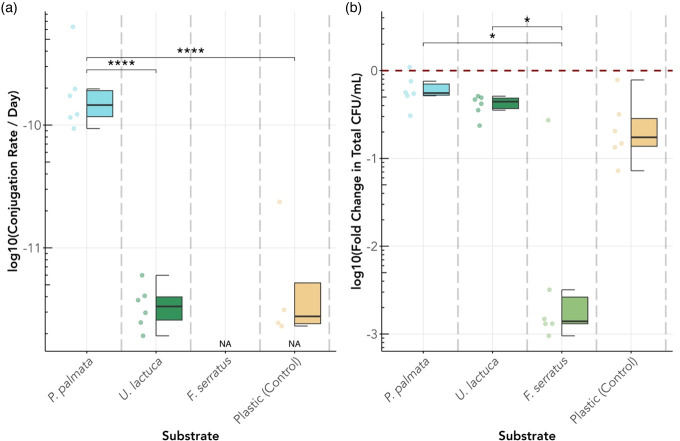
*E. coli* survival and conjugation in the presence of different seaweed substrates. (a) *E. coli* conjugation rates (transconjugants/donors×recipients×time) and (b) fold change in population densities after 48 h in seawater in the presence of one of three desiccated seaweeds or a plastic control. Asterisks denote a significant difference between treatments, *P<0.05; ****P<0.0001. For all statistical model values, see Tables S2 and S3. For absolute *E. coli* densities, see Fig. S2. NA – transconjugant numbers below the limit of detection in at least one replicate.

To test whether pKJK5::gfp conjugation rate differed significantly between treatments, we compared a model including treatment as a fixed effect to a null model. The model fit improved significantly with substrate as a predictor (likelihood ratio test, *χ*^2^=10.5, *P*=2.2×10^−16^; Fig. 1a), indicating a strong effect of substrate identity on conjugation rate. Conjugation rates in the presence of a plastic control or *U. lactuca* remained low (1.83×10^−13^) and were not significantly different from each other (pairwise EMM contrast, *t*=13, *P*=0.78). Conjugation remained below the limit of detection (10^−4^ transconjugants per ml) in the presence of *F. serratus* and could therefore not be compared to other groups. In contrast, conjugation rate was ~100-fold higher in the presence of *P. palmata* than for the other two seaweeds and plastic control (Fig. 1a; pairwise EMM contrasts, *P*<0.0001), reaching a rate similar to that observed during high-nutrient conditions (3.37×10^−12^; Fig. S1). Our measured conjugation rates were accompanied by changes in *E. coli* density throughout the experiment, which means our absolute conjugation rate values are likely inaccurate and should not be compared to literature values. Nevertheless, relative differences in conjugation between treatments were robust and were also reflected in population ratios of transconjugant fraction (Methods; Table S1).

To further explore how conjugation rate in the presence of different biotic substrates can be explained by differences in * E. coli* growth or survival, we next assessed the fold change in *E. coli* density after 48 h of incubation (Fig. 1b) . *E. coli* densities showed a consistent decrease from ~4.4×10^7^ down to ~10^4^–10^7^ c.f.u. ml^−1^ (Fig. S2) after 2 days of incubation with substrate identity having a significant effect (Kruskal–Wallis test, *K*=15.2, *df*=3, *P*=0.0017). The presence of *F. serratus* led to an 18.4-fold decrease in *E. coli* density (Wilcoxon paired tests, *P*<0.05), explaining the lack of detectable conjugation in this treatment (Fig. 1a) – any conjugation rate lower than ~3.4×10^−8^ could not be detected with these low final *E. coli* densities (see Methods). Exposure to *P. palmata* and *U. lactuca* led to a modest (up to ~fourfold) drop in *E. coli* density, which was not significantly different between treatments (Wilcoxon paired test, *V*=31, *P*=0.062; Fig. 1b). However, conjugation rate differed significantly between these two species (pairwise EMMs contrast, *t*=13, *P*=2.86×10^−6^; Fig. 1a), with *P. palmata* promoting conjugation independently of *E. coli* density and survival.

## Discussion

There is an increasing appreciation of the fact that biotic interactions can influence the rate of plasmid conjugation and other aspects of plasmid biology (e.g. plasmid fitness and maintenance) [29,35]. Such effects could have implications for the evolution and epidemiology of horizontally acquired antimicrobial resistance in the natural environment, where human-associated bacteria encounter a wide range of different organisms with which they can interact. Coastal areas serve as a relevant test case, as sewage pollution increasingly brings into contact gut bacteria with seaweeds, which are known to produce a wide variety of secondary metabolites which could affect processes such as biofilm formation or stress responses known to influence rates of conjugation [36,37].

We here show that conjugation rates between and survival of *E. coli* can be influenced by the presence of seaweed. On the one hand, the edible red seaweed species dulse *P. palmata* resulted in a marked increase of IncP plasmid conjugation rate in comparison with a plastic control. This result, which was not due to cell density increases, stands in contrast to the no-seaweed high-nutrient control treatment, where a high observed conjugation rate mirrored an increase in *E. coli* density. We hypothesize that this conjugation-promoting effect could be due to *P. palmata* secondary metabolites, potentially in conjunction with additional or synergistic effects of the native seaweed microbiome. On the other hand, the presence of serrated wrack *F. serratus* led to a ~100-fold drop in *E. coli* density and was accompanied by a complete lack of observable conjugation events. While we did not assess the reasons for *E. coli* density decrease throughout our experiments, this could be due to a lack of nutrients, salt stress or biotic competition with a background community in unsterilized seawater.

We add to the scant evidence that inter-species interactions can influence the rate of bacterial HGT, which is an area that offers many avenues for future research, including elucidating molecular mechanisms, quantifying effects imposed by different species and measuring ecosystem effects. The findings presented here could also have implications for understanding and managing microbial pollution in coastal environments. First, some seaweeds (in this case *F. serratus*) could offer potential to reduce the load of suspended faecal bacteria. Second, AMR risks can be underestimated when considering *E. coli* density but not variation in HGT rates across environments (in this case *P. palmata*). Third, although plastics in aquatic ecosystems have been identified as a hotspot for HGT [38], our results indicate that biotic surfaces could be even more important.

## Supplementary material

10.1099/mic.0.001622 Supplementary Material 1.

10.1099/mic.0.001622 Supplementary Data Sheet 1.

## Abbreviations

AMR: antimicrobial-resistant
EMM: estimated marginal mean
HGT: horizontal gene transfer
LB: Luria-Bertani
