# Supplementary material for: Seaweed exposure modulates Escherichia coli plasmid conjugation rate
Source: Microbiology (Reading). 2025 Oct 14;171(10):001622. doi: 10.1099/mic.0.001622 (PMC13293327; doi:10.1099/mic.0.001622)
Supplement: Supplementary Material 1. [file mic-171-01622-s001.pdf]

Supplementary Material to:

Seaweed exposure modulates *E. coli* plasmid conjugation rate

David Sünderhauf[†1]*, Macaulay Winter[†1,2,3], Jasmine Ramshaw[1,2,4,5], Emily M Stevenson[1,2,6], Michiel Vos[1,2]

1= Environment and Sustainability Institute, University of Exeter, Penryn, Cornwall, TR10 9FE, United Kingdom

2= European Centre for Environment and Human Health, University of Exeter Medical School, Environment and Sustainability Institute, Penryn Campus, TR10 9FE, UK

3=Department of Biological Sciences, Clemson University, Clemson, South Carolina, USA

4= The Marine Biological Association of the UK, The Laboratory, Citadel Hill, Plymouth PL1 2PB, United Kingdom

5= School of Biological and Marine Sciences, University of Plymouth, Plymouth, Devon, PL4 8AA, United Kingdom

6= Marine Ecology & Society, Plymouth Marine Laboratory, Prospect Place, West Hoe Plymouth, PL1 3DH

[†]= shared first author

*= corresponding author: d.sunderhauf@exeter.ac.uk; david@sunderhauf.net

Supplementary Figures:

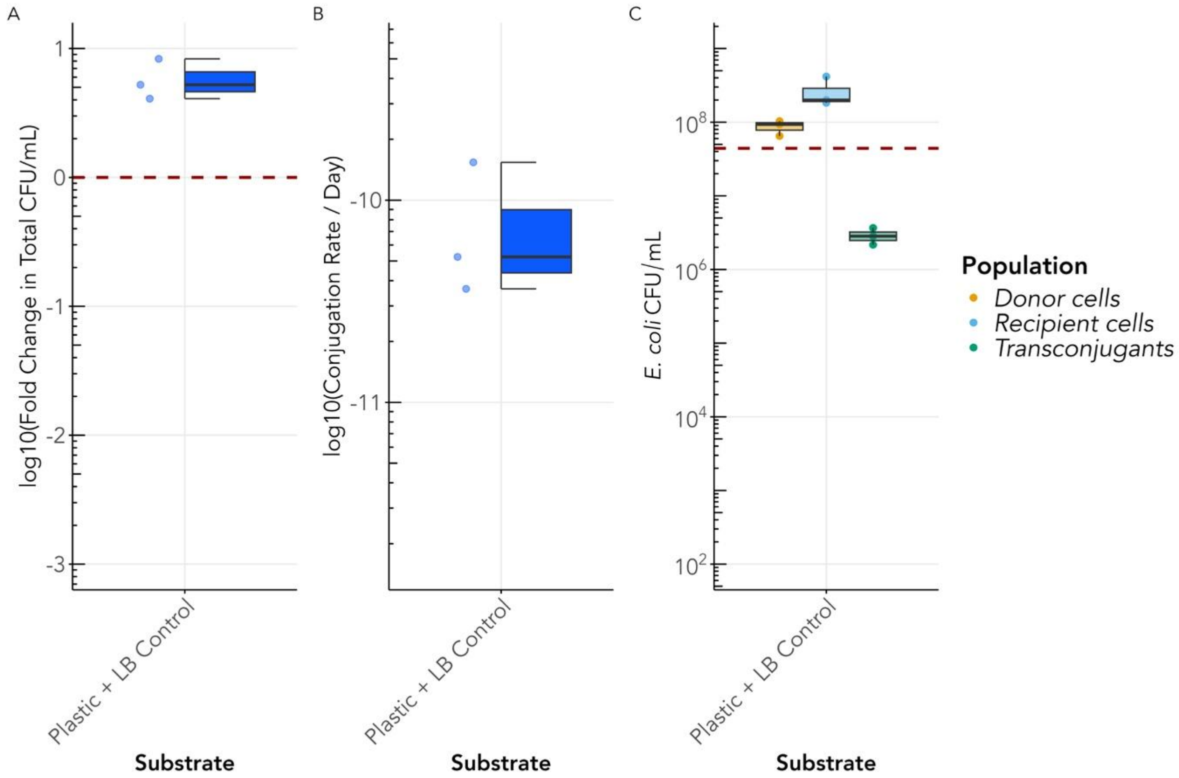

<u>Figure S1: Sterile high-nutrient conditions show potential for detecting conjugation in our model system.</u>

Fold change in *E. coli* population densities (A), conjugation rate (B), and CFU/mL of E. coli donor, recipient, and transconjugant cells (C) after 48 hours in the presence of sterile plastic in LB broth. The dashed red line in C signifies the *E. coli* input CFU/mL at $t = 0$.

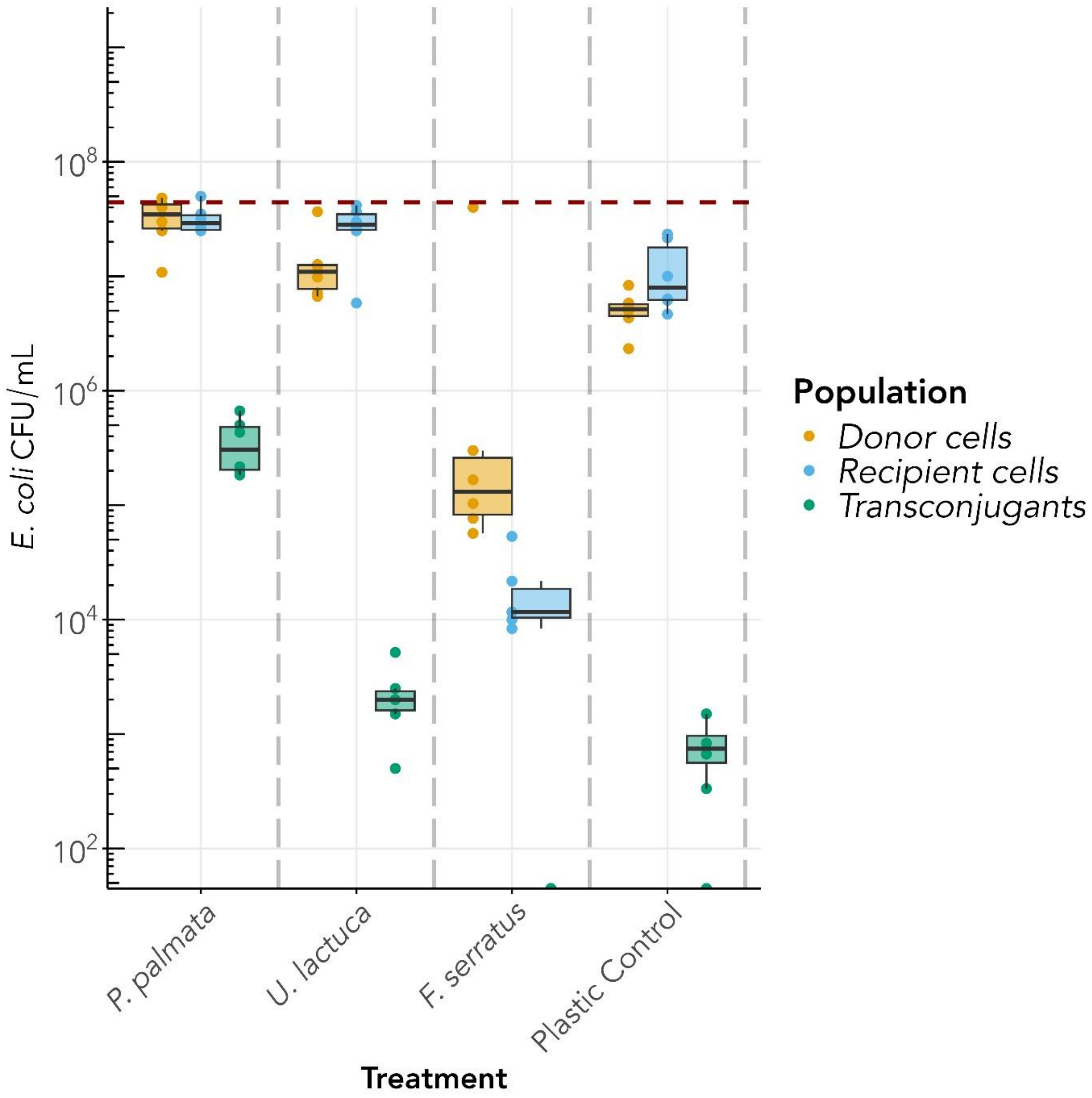

Figure S2: E. coli CFU/mL after conjugation in seawater with seaweed substrates.

CFU/mL of E. coli donor, recipient, and transconjugant cells after 48 hours in the presence of desiccated seaweed or a plastic control in unsterilised seawater. The dashed red line signifies the E. coli input CFU/mL at t = 0.

Supplementary Tables:

Table S1. Average conjugation rate vs transconjugant formation population ratio.

**Treatment**	**Conjugation rate** [transconjugants / donors * recipients * time]	**Transconjugant fraction** [percentage of recipients that became transconjugants]
P. palmata	2.22×10^{-10}	1.1%
U. lactuca	3.52×10^{-12}	0.0082%
F. serratus	*below detection limit*	*below detection limit*
Plastic control	5.26×10^{-12}	0.0056%

Table S2. Estimated marginal means comparisons of log10 conjugation rates with *p* values adjusted using FDR correction (Figure 1A)

Substrate 1	Substrate 2	estimate	SE	df	t ratio	*p* value	Adjusted *p* value
U. lactuca	Plastic Control	-0.1375259	0.2036879	13	-0.6751795	0.7816577	0.7816577
P. palmata	Plastic Control	1.5871558	0.2036879	13	7.7920966	8.342192e-06	1.251329e-05
P. palmata	*U. lactuca*	1.7246817	0.1821840	13	9.4667024	9.537520e-07	2.861256e-06

Table S3. Wilcoxon paired tests for the density change between treatments (Figure 1B)

Substrate 1	Substrate 2	n1	n2	statistic	*p* value	Adjusted *p* value
P. palmata	*U. lactuca*	6	6	31	0.041	0.062
P. palmata	*F. serratus*	6	6	36	0.002	0.013
P. palmata	Plastic Control	6	6	30	0.065	0.078

U. lactuca	*F. serratus*	6	6	35	0.004	0.013
U. lactuca	Plastic Control	6	6	29	0.093	0.093
F. serratus	Plastic Control	6	6	4	0.026	0.052
